# Astaxanthin Promotes Nrf2/ARE Signaling to Alleviate Renal Fibronectin and Collagen IV Accumulation in Diabetic Rats

**DOI:** 10.1155/2018/6730315

**Published:** 2018-03-21

**Authors:** Xiaoyu Zhu, Yongjun Chen, Qing Chen, Huiyuan Yang, Xi Xie

**Affiliations:** ^1^Key Laboratory of Tropical Biological Resources of Ministry of Education, Hainan University, Haikou, China; ^2^Hainan Provincial Key Laboratory for Tropical Hydrobiology and Biotechnology, College of Marine Science, Hainan University, Haikou, Hainan, China; ^3^State Key Laboratory of Marine Resource Utilization in South China Sea, Hainan University, Haikou, China; ^4^College of Materials and Chemical Engineering, Hainan University, Haikou, China; ^5^School of Life Science, Institute of Tropical Agriculture and Forestry, Hainan University, Haikou, China

## Abstract

Astaxanthin (AST), a natural keto-carotenoid classified as a xanthophyll, is well known for its antioxidant properties. AST can ameliorate the pathological characteristics of diabetic nephropathy (DN). However, the underlying mechanisms remain to be explored. This study was aimed at exploring whether AST exerts a protective effect on DN via activating nuclear factor erythroid 2-related factor 2– (Nrf2–) antioxidative response element (ARE) signaling. Streptozotocin-induced diabetic rats were treated with AST for 12 weeks. We found that AST treatment ameliorated renal morphological injury. Reduced fibronectin and collagen IV protein expression were found in the kidneys of diabetic rats. Furthermore, AST promoted the nuclear translocation of Nrf2 and increased its downstream protein heme oxygenase-1 and superoxide dismutase 1 expression. AST also increased the activity of SOD and decreased malondialdehyde generation in the serum of diabetic rats. These results suggest that the renoprotective effect of AST on DN partly depends on Nrf2–ARE signaling. The antioxidative stress effect of AST is responsible for the activation of Nrf2–ARE signaling in DN.

## 1. Introduction

Diabetic nephropathy (DN), one of the most serious microvascular complications of diabetes mellitus, is the leading cause of end-stage renal failure [1]. The WHO claims that there are 422 million diabetic patients all over the world, and approximately one third of them are affected by DN [2, 3]. The pathological progress of DN is sequential, which includes basement membrane thickening, accumulation of extracellular matrix (ECM) production such as fibronectin (FN) and collagen IV (Col IV), glomerulosclerosis, and interstitial fibrosis [4]. ECM accumulation, the pathological hallmark of DN, is caused by a complex interplay of numerous factors [5]. Although the pathogenesis of DN is complex, hyperglycemia-mediated oxidative stress is still considered as the pivotal factor in the initiation and development of DN [6]. Excessive reactive oxygen species (ROS) induced by hyperglycemia is associated with multiple signaling that leads to renal damage [7, 8]. ROS, a class of oxygen-derived molecules with unpaired electrons or atoms, including superoxide anions and hydrogen peroxide, exerts strong chemical reactions. The generation of oxidative stress is due to the amount of ROS exceeding the amount of oxidant scavengers and the abnormal ratio of NADPH/NADP^+^. Many studies have suggested that the elevated levels of oxygen-derived free radicals and the increase in ROS induced by diabetes mellitus can cause damage to cellular components [9]. Glucose activates aldose reductase and polyol signaling in renal cells under high-glucose conditions, which decreases the ratio of NADPH/NADP^+^. Elevated glucose induces the de novo synthesis of diacylglycerol, which activates protein kinase C (PKC) signaling and increases NADPH oxidases in mitochondria, ultimately leading to mesangial expansion and thick basement membrane in the glomerular mesangial cells [10].

Nuclear factor erythroid 2-related factor 2 (Nrf2), a redox-sensitive transcription factor, is bound to the negative regulator Kelch-like ECH-associated protein 1 (Keap1) in the cytoplasm and degraded by the ubiquitin–proteasome pathway in basal condition [11]. Once oxidative stress occurs, Nrf2 dissociates from Keap1 and translocates to the nucleus, where it functions as a strong activator of the antioxidant response element (ARE) and regulates protective genes, such as heme oxygenase-1 (HO-1) and superoxide dismutase 1 (SOD1), eventually enhancing cell survival [12]. As a pivotal signaling pathway that suppresses oxidative stress and maintains the balance of intracellular redox, Nrf2–ARE has been wildly explored in DN and other diabetic complications [13]. A high ROS level was observed in Nrf2 knockout mice, which resulted in renal injury and DNA damage [14]. Nrf2 activators sulforaphane and cinnamic aldehyde improved kidney performance and ameliorated pathological alterations in streptozotocin- (STZ-) induced diabetic mice [15]. Bardoxolone methyl, another activator of Nrf2, rapidly improved estimated glomerular filtration in type 2 diabetes patients [16]. Nrf2 also exhibited protective effects in diabetic retinopathy and diabetic neuropathy by suppressing oxidative stress [17–19]. Thus, we believe that activating Nrf2 signaling is beneficial in preventing or delaying the progression of DN, at least in part, by diminishing the excessive ROS induced by high glucose.

Astaxanthin (AST) is a nontoxic and organic fat-soluble xanthophyll carotenoid [20]. It is found in various marine organisms, such as algae, shrimps, and salmon. The USFDA has approved AST as a nutraceutical in 1999 [21]. AST supplementation is beneficial for the eyes, skin, and heart [22]. The antioxidative potency of AST is reportedly 800 times stronger than coenzyme Q10 and 6000 times stronger than vitamin C [23]. Aside from the remarkable antioxidative performance, AST has been found to have antidiabetes, anti-inflammation, cardiovascular disease prevention, and anticancer properties [23, 24]. Studies have confirmed that the antioxidant effect of AST is closely related to Nrf2–ARE signaling [25–27]. Moreover, AST has been reported to improve renal function in diabetic rats and reduce renal cell injury [20, 28, 29]. AST significantly reduces the amount of malondialdehyde (MDA) in plasma and partly reverses glomerular hypertrophy and tubular expansion in diabetic rats [20]. After long-term treatment with AST, a notable decrease in blood glucose and urinary albumin levels and DNA damage were observed in diabetic mice [28]. Additionally, AST accumulated in the mitochondria of human mesangial cells, where it suppressed the high-glucose-induced ROS production [29]. However, whether AST can enhance the resistance to oxidative stress through Nrf2–ARE signaling and then alleviate DN remains to be clearly defined.

This work was aimed at determining whether AST could alleviate the pathological progress of DN by activating Nrf2–ARE signaling and eventually diminish the excessive oxidative stress and FN and Col IV accumulation in diabetic kidneys.

## 2. Materials and Methods

### 2.1. Reagents and Antibodies

STZ was purchased from Sigma-Aldrich, (St. Louis, MO, USA). AST was obtained from Boster Biological Technology Co. (Wuhan, China). The antibody against Nrf2 was from Santa Cruz Biotechnology Co. (Santa Cruz, CA, USA). Antibodies against Col IV, FN, SOD1, HO-1, and Keap1 were from Boster Biological Technology Co. (Wuhan, China). Antibodies against Histone H3, *β*-actin, and horseradish peroxidase conjugated secondary antibodies were obtained from Beyotime Biological Technology (Haimen, China). ECL Plus Kit and BCA Protein Assay Kit were obtained from Beyotime Biological Technology Co. (Haimen, China).

### 2.2. Animal Experiment

Male Sprague-Dawley rats (*n* = 50, 200 ± 10 g) were supplied by the Experimental Animal Center, Sun Yat-Sen University, Guangzhou, China. All animal procedures conformed to the China Animal Welfare Legislation and were reviewed and approved by the Sun Yat-Sen University Committee on Ethics in the Care and Use of Laboratory Animals (Permit Number: 20150603005; Animal Quality Certificate No.: 0007630). All animals were housed under standard conditions with food and water freely obtained. After being fed with a regular diet for 1 week, they were assigned to a diabetic model group (*n* = 34), which were fed with a high-fat, high-glucose diet for the following 4 weeks, a normal control group (*n* = 8), which were fed a normal diet, and a vehicle group (*n* = 8). After 4 weeks of feeding, diabetic model group rats were given a single intraperitoneal injection of STZ on 30 mg/kg, freshly prepared. The normal group and vehicle group rats were injected with an equal volume of citrate buffer. Diabetic rats were accepted by the fasting blood glucose measurement ≥11.1 mmol/L after a 72 h injection. Diabetic rats were randomized into an administration group (*n* = 8) to receive AST (25 mg/kg daily i.g.), and the other diabetic rats and vehicle group rats received an equal volume of olive oil. Rats were sacrificed after a 12-week treatment. The blood sample was collected from the abdominal vein, and serum was obtained by centrifugation at 3000*g* for 15 min and stored at −80°C. Kidney cortex samples were rapidly excised, frozen in liquid nitrogen quickly, and then stored at −80°C or fixed in 10% neutral buffered formalin.

### 2.3. Biochemical and Morphological Studies

Blood glucose, blood urea nitrogen, serum creatinine, and urine protein were analyzed in Hainan General Hospital. Blood urea nitrogen was tested with Roche UREAL (Roche Ltd., Switzerland), serum creatinine was measured with Roche CREJ2 (Roche Ltd., Switzerland), and urine protein was determined with Szybio TP (Shenzhiyuan Biological Technology, Wuhan, China) according to the manufacturer's instructions. They were then analyzed with the Roche cobas 8000 modular analyzer (Roche Ltd., Switzerland). The cortex of the kidneys was separated and fixed in 10% formaldehyde before being embedded in paraffin. Then, 4 *μ*m thick sections were stained with periodic acid–Schiff (PAS) or hematoxylin–eosin (HE). The cross section yielding the maximum diameter of the glomerulus was photographed. Glomerular tuft areas were analyzed with Image-Pro Plus. Forty glomeruli were chosen from three slides in each animal randomly. The mesangial matrix index (MMI, in %) was calculated as the ratio of the mesangial area to the glomerular area × 100.

### 2.4. Western Blot Assay

In total, 15 mg of frozen kidney tissues was lysed in 0.2 mL of commercial RIPA solution containing 1 mM of PMSF. After centrifugation at 14,000*g* for 15 min, the supernatants were collected. The protein concentrations were measured with the BCA Protein Assay Kit. The nuclear and cytoplasmic proteins were extracted using a commercial kit according to the instructions.

Samples containing 60 *μ*g of total proteins or 30 *μ*g of nuclear proteins were loaded and separated in 8%, 10%, and 12% sodium dodecyl sulfate–polyacrylamide gel electrophoresis and transferred to NC membranes. The membranes were blocked with 5% skim milk in TBST (pH = 7.4) for 1 h and incubated with primary antibodies at 4°C. The membranes were incubated with corresponding secondary antibodies for 1 h and then reacted with ECL Plus Substrate. Chemiluminescence signals were measured by using X-ray films, and then the films were scanned using HP LaserJet Professional M1213nf MFP. Densitometry was quantified and analyzed by image processing software ImageJ.

### 2.5. Statistical Analysis

The data were analyzed using SPSS 22.0 software. Values were expressed as means ± SD. Statistical analyses of data were performed by one-way ANOVA using post hoc multiple comparisons. A *P* value less than 0.05 was considered to be statistically significant. All experiments were performed at least three times.

## 3. Results

### 3.1. Effects of AST on Metabolic Parameters in STZ-Induced Diabetic Rats

**Figure 1:**
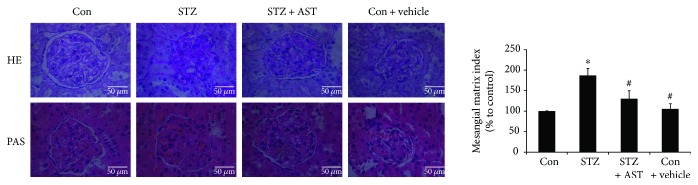
Glomerular injury in the kidneys of STZ-induced diabetic rats. Glomerular histopathology was assessed by PAS and HE staining. The images display the representative sections at an original magnification of ×400. The MMI represented the ratio of the mesangial area to the glomerular area × 100. ^∗^*P* < 0.01 versus control group and ^#^*P* < 0.05 versus diabetic group. Scale bar represents 50 *μ*m.

As shown in Table 1, kidney weight and kidney hypertrophy index (KW/BW), fasting blood glucose, blood urea nitrogen, serum creatinine, and urine protein over 24 h were significantly increased in STZ-induced diabetic rats compared with those in control rats (*P* < 0.05, Table 1). After 12 weeks of treatment with AST (25 mg/kg daily), the diabetic rats exhibited a significant reduction in these parameters except blood glucose (*P* < 0.05, Table 1). The representative PAS- and HE-stained glomeruli are shown in Figure 1. The PAS-positive matrix in the glomeruli was increased in diabetic rats compared with control rats; however, treatment with AST reversed this change. The MMI was calculated to evaluate the effect of AST on matrix accumulation. AST significantly reduced the MMI in the glomeruli of diabetic rats (*P* < 0.05, Figure 1). HE staining showed that the kidney of diabetic rats exhibited structural damage, glomerular hypertrophy, mesangial hyperplasia, and basement membrane thickening. Treatment with AST significantly improved the pathological changes in the kidney of diabetic rats (Figure 1).

### 3.2. AST Increased Serum SOD Activity and Reduced MDA Content in Diabetic Rats

**Figure 2:**
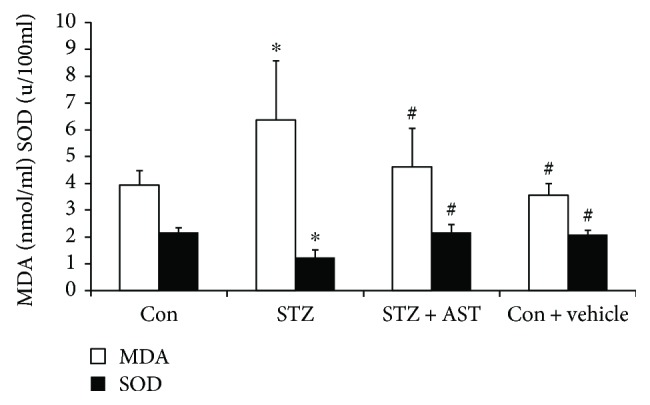
Effects of AST on serum MDA content and SOD activity in STZ-induced diabetic rats. ^∗^*P* < 0.01 versus the control group and ^#^*P* < 0.05 versus the STZ-induced diabetic group.

To evaluate the effect of AST on preventing oxidative damage of the kidney, MDA content and SOD activity were explored. After treatment with AST for 12 weeks, the serum MDA level was significantly reduced in diabetic rats. Meanwhile, SOD activity was increased (*P* < 0.05, Figure 2).

### 3.3. AST Promoted the Activation of Nrf2–ARE Signaling in Diabetic Rats

**Figure 3:**
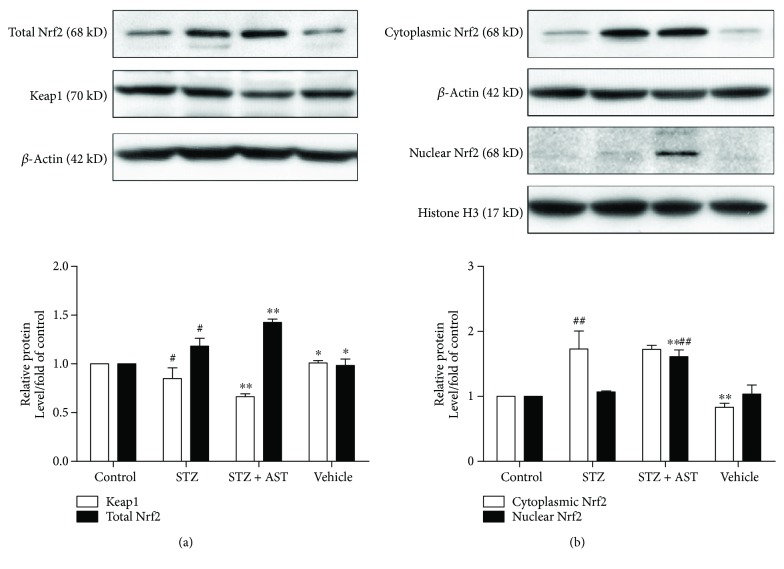
Effects of AST on the activation of Nrf2–ARE signaling. (a) AST obviously increased the total Nrf2 expression and decreased the total Keap1 levels in the kidney of diabetic rats. (b) AST further increased the nuclear Nrf2 levels in the kidney of diabetic rats. ^∗^*P* < 0.05 and ^∗∗^*P* < 0.01 versus the STZ-induced diabetic group; ^#^*P* < 0.05 and ^##^*P* < 0.01 versus the control group.

The Keap1–Nrf2–ARE pathway is one of the most essential endogenous antioxidative systems that respond to various attacks, including oxidative damage. Once endogenous stress occurs, Nrf2 dissociates from its cytoplasmic repressor Keap1 and translocates into the nucleus to bind with ARE. Our research showed that the diabetic group exhibited a declining trend of the Keap1 protein level in the kidney. However, a low Keap1 protein level was observed in the AST-treated group. Corresponding with these results, the total Nrf2 protein level increased in the kidney of diabetic rats, and this trend was enhanced after treatment with AST (*P* < 0.05, Figure 3(a)). Furthermore, a slight increase in the Nrf2 level was observed in the nuclei of diabetic rats, and AST sharply increased the nuclear Nrf2 levels (*P* < 0.05, Figure 3(b)). These results showed no significant difference between the vehicle and control groups. Our data suggest that AST increases the activation of Nrf2–ARE antioxidative signaling by promoting the nuclear translocation of Nrf2.

### 3.4. AST Increased the Protein Expression of HO-1 and SOD1 in the Kidney of Diabetic Rats

**Figure 4:**
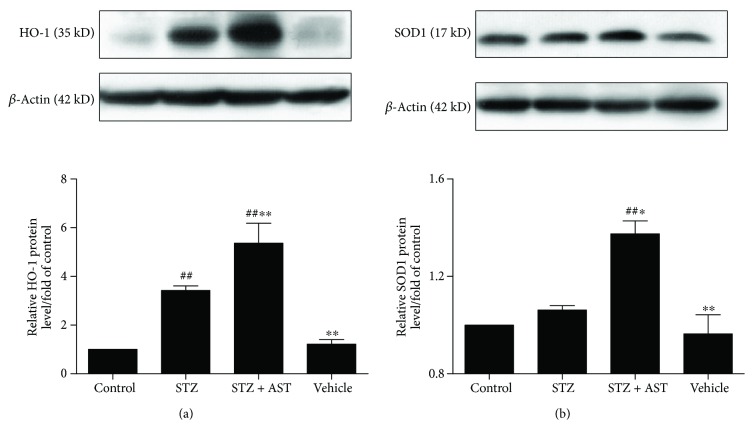
Effects of AST on HO-1 and SOD1 expression. (a) AST treatment obviously increased HO-1 protein levels in the kidney of diabetic rats. (b) AST treatment increased the protein expression of SOD1 in the kidney of diabetic rats. ^∗^*P* < 0.05 and ^∗∗^*P* < 0.01 versus the STZ-induced diabetic group; ^#^*P* < 0.05 and ^##^*P* < 0.01 versus the control group.

HO-1 and SOD1 are genes regulated by Nrf2 [12]. Western blot showed that protein levels of HO-1 in the kidney were significantly upregulated by AST treatment in diabetic rats (Figure 4(a)). AST also significantly enhanced the protein levels of SOD1 in the kidney (Figure 4(b)). These results were consistent with the findings of Nrf2 and showed no significant difference between the vehicle and control groups. Our data confirmed that Nrf2 translocated into the nuclei and then activated the expression of downstream genes to exert its antioxidative effect.

### 3.5. AST Decreased the Protein Expression of FN and Col IV in the Kidney of Diabetic Rats

**Figure 5:**
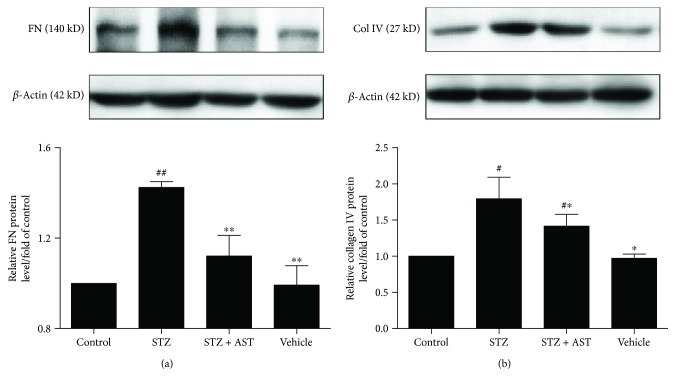
Effects of AST on the expression of FN and Col IV. (a) AST treatment significantly decreased FN levels in the kidney of diabetic rats. (b) AST treatment decreased the protein expression of Col IV in the kidney of diabetic rats. ^∗^*P* < 0.05 and ^∗∗^*P* < 0.01 versus the STZ-induced diabetic group; ^#^*P* < 0.05, ^##^*P* < 0.01 versus control group.

Excessive accumulation of ECM causes basement membrane thickening and expansion of the glomerular mesangial matrix and tubulointerstitial space [30]. FN and Col IV are the major components of the ECM. Compared with normal rats, diabetic rats showed a sharp increase in the protein content of FN and Col IV. After treatment with AST for 12 weeks, the protein expression of FN and Col IV was significantly decreased (*P* < 0.05, Figure 5). These results showed no significant difference between the vehicle and control groups and suggested that AST can ameliorate the pathological process of DN by alleviating the accumulation of ECM components.

## 4. Discussion

DN is one of the most serious microvascular complications of diabetes mellitus. The most important characteristic of DN is the accumulation of ECM [30, 31]. Therefore, depressing the excessive generation of ECM induced by hyperglycemia plays an important role in the prevention and treatment of DN. In this study, FN and Col IV, the major components of ECM, were induced in diabetic rats, whereas AST reversed these pathological changes. Morphologically, excessive accumulation of the glomerular mesangial matrix and structural damage were observed in diabetic rats. However, AST treatment ameliorated these changes. These results indicate that AST can interfere with the pathogenesis of DN by reducing the accumulation of ECM components, which is consistent with previous studies [14, 30].

Excessive ROS induced by hyperglycemia is involved in a variety of signaling pathways, such as PKC signaling, polyol signaling, and hexosamine signaling, that lead to complications of diabetes [32]. Moreover, ROS can induce the generation of growth factors and cytokines, which will enhance renal cell hypertrophy or proliferation by upregulating ECM expression and decreasing their degradation, eventually causing renal fibrosis [33, 34]. Therefore, eliminating excessive ROS production is pivotal in the treatment of DN. In diabetes, the activity of antioxidant enzymes decreases, decreasing the ability to scavenge free radicals. The activity of SOD in serum indicates the ability to scavenge oxygen-free radicals. The content of MDA, the end product of lipid peroxidation, reflects the severity of free radical attack. In the current study, the serum MDA content increased in diabetic rats, and the activity of SOD decreased. However, these phenomena were reversed after AST treatment. These findings suggest that AST can enhance the antioxidative ability and ameliorate oxidative stress in diabetic rats.

ARE is a *cis*-regulatory element with specific DNA sequences that are present in the upstream regulatory regions of genes encoding a series of detoxifying enzymes such as HO-1 and SOD1 [4]. Once insulted by oxidative stress, cells exert adaptive activation of Nrf2, thereby inducing ARE-dependent gene expression to protect themselves from oxidative injury. In the kidney of diabetic rats, the slightly increased nuclear Nrf2 content, decreased Keap1, and HO-1 upregulation could be considered as an adaptive response to oxidative stress. Considering the excessive expression of FN and Col IV in the kidney of diabetic rats, this kind of adaptive response may not be strong enough to prevent the development of DN. In the AST-treated group, high SOD1, HO-1, and nuclear Nrf2 protein levels were observed, which suggest activation of Nrf2–ARE signaling. Nrf2 agonists work by suppressing Keap1, the inhibitor of Nrf2 [35]. By silencing Keap1 with siRNA, the production of ECM in human renal mesangial cells was reduced [15]. In the current study, we also found that the protein level of Keap1 was markedly decreased after AST treatment. All these findings suggest that AST can enhance the expression level of renal antioxidant enzymes and intervene in the pathological process of DN, at least in part, due to the activation of Nrf2–ARE signaling.

In summary, the present study demonstrated that AST increased the nuclear content of Nrf2 and HO-1 and SOD1 protein expression and decreased the accumulation of FN and Col IV, therefore delaying the pathological process of DN. In addition, AST elevated the antioxidant ability of diabetic rats and ameliorated lipid peroxidation. This study mainly explored the protective effects and mechanisms of AST on DN in vivo, and follow-up studies will further clarify the molecular mechanisms of AST on Nrf2–ARE in vitro. We will also investigate whether AST can exert an antioxidative effect through other signaling pathways to support the clinical application of AST as a therapeutic drug for DN.

## Figures and Tables

**Table 1:** Effects of AST on renal metabolic and biochemical parameters in STZ-induced diabetic rats.

Parameter	Control (*n* = 8)	STZ (*n* = 8)	STZ + AST (*n* = 8)	Control + vehicle (*n* = 8)
Body weight (g)	474.36 ± 9.45	228.47 ± 17.12^∗^	329.73 ± 23.91^∗^^,#^	486.12 ± 7.56^#^
Kidney weight (g)	2.30 ± 0.13	3.49 ± 0.61^∗^	2.93 ± 0.37^∗^	2.19 ± 0.11^#^
KW/BW (%)	0.49 ± 0.06	1.27 ± 0.12^∗^	0.78 ± 0.20^∗^^,#^	0.45 ± 0.04^#^
Blood glucose (mM)	5.02 ± 0.49	23.9 ± 2.91^∗^	21.7 ± 1.81^∗^	5.78 ± 0.35^#^
BUN (mM)	5.98 ± 1.01	14.02 ± 3.20^∗^	9.98 ± 1.84^#^	6.08 ± 0.98^#^
Cr (*μ*M)	27.81 ± 3.98	42.56 ± 9.21^∗^	28.34 ± 8.92^#^	29.01 ± 4.76^#^
UP (mg/24 h)	13.98 ± 3.29	98.89 ± 29.98^∗^	69.79 ± 21.34^∗^^,#^	14.32 ± 1.36^#^

BUN: blood urea nitrogen; Cr: serum creatinine; UP 24 h: urine protein for 24 hours. Data are means ± SD; *n* = 8. ^∗^*P* < 0.01 versus control group and ^#^*P* < 0.05 versus STZ-diabetic group.
